# The implication of next-generation sequencing in the diagnosis and clinical management of non-Hodgkin lymphomas

**DOI:** 10.3389/fonc.2023.1275327

**Published:** 2023-11-08

**Authors:** Victor Tomacinschii, Adrian Mosquera Orgueira, Carlos Aliste Santos, Maria Robu, Sanda Buruiana, Maximo Francisco Fraga Rodriguez

**Affiliations:** ^1^Department of Hematology, Nicolae Testemitanu State University of Medicine and Pharmacy, Chisinau, Moldova; ^2^Department of Hematology, Public Medical Sanitary Institution (PMSI) Institute of Oncology, Chisinau, Moldova; ^3^University Hospital of Santiago de Compostela, Servizo Galego de Saude (SERGAS), Santiago de Compostela, Spain; ^4^Health Research Institute of Santiago de Compostela, Santiago de Compostela, Spain; ^5^ Department of Forensic Sciences, Pathology, Ginecology and Obstetrics and Pediatrics, Faculty of Medicine, University of Santiago de Compostela, Santiago de Compostela, Spain

**Keywords:** lymphomas, non-Hodgkin lymphomas, genomics, diagnosis, next-generation sequencing

## Abstract

Next generation sequencing (NGS) is a technology that broadens the horizon of knowledge of several somatic pathologies, especially in oncological and oncohematological pathology. In the case of NHL, the understanding of the mechanisms of tumorigenesis, tumor proliferation and the identification of genetic markers specific to different lymphoma subtypes led to more accurate classification and diagnosis. Similarly, the data obtained through NGS allowed the identification of recurrent somatic mutations that can serve as therapeutic targets that can be inhibited and thus reducing the rate of resistant cases. The article’s purpose is to offer a comprehensive overview of the best ways of integrating of next-generation sequencing technologies for diagnosis, prognosis, classification, and selection of optimal therapy from the perspective of tailor-made medicine.

## 1 Introduction

Non-Hodgkin’s lymphomas (NHL) are hematopoietic tumors that develop from the malignant proliferation of the lymphatic tissue. NHLs are the most common hematological neoplasms, accounting for roughly 3% of cancer cases worldwide. According to the most recent GLOBOCAN data, 544,352 new cases of NHL were diagnosed worldwide in 2020 (1, 2).

Classifications of non-Hodgkin’s lymphomas have undergone numerous refinements and completions over time, ranging from classifications based on the histological and immunological profile of tumors (Rappoport classification (3), Kiel classification (4), Lukes and Collins classification (5), etc.) to current classifications systems (World Health Organization Classifications from 2016 and 2022 and The International Consensus Classification of Mature Lymphoid Neoplasms (6–8)) which divides non-Hodgkin’s lymphomas according to histological, immunohistochemical and gene expression profiles (GEP).

Healthcare practitioners prefer also to group NHL subtypes based on the speed of disease progression. For example, indolent B-cell lymphomas, such as follicular lymphomas, marginal zone lymphoma, small lymphocytic lymphoma, proceed as chronic incurable diseases, in which the clinical course is slow-progressing and oligosymptomatic for a long period of time but for the treatment of which, however, regular exposure to toxic cytostatic drugs and/or radiation therapy is required (9).

On the other hand, aggressive B-cell lymphomas represent a heterogeneous category of lymphomas that may involve precursor lymphoid neoplasms (B-lymphoblastic leukaemia/lymphoma NOS and B-lymphoblastic leukaemia/lymphoma with recurrent genetic abnormalities) as well as a variety of mature B-cell lymphomas, like Burkitt lymphoma, mantle cell lymphoma, primary effusion lymphoma, and diffuse large B-cell lymphoma. They have aggressive behavior, with frequent extranodal involvement and require immediate treatment, otherwise, resulting in patient’s rapid desmise. Although modern treatment regimens can increase survival in certain patients with aggressive large-cell lymphomas (approximately 60% in diffuse B-large-cell lymphoma, about 30% in peripheral T-cell lymphomas), disease progression remains the leading cause of death (1, 9–11).

The notable progress in recent years should be attributed to the advances in molecular genetics. These advancements have enabled a shift from analyzing individual genes and markers to conducting comprehensive studies on multiple genes or their expressed products concurrently, particularly in the context of cancer research (12–14). The emergence of high-tech genome-wide research methods and their integration into publicly available databases make it possible to obtain more detailed information about the mechanisms of oncogenesis, explain the division of tumors by histological types, differentiate gene networks that determine the main stages of tumor pathogenesis, and study the mechanisms of drug resistance (15–18).. The study of gene expression profiles in certain types and subtypes of tumors makes it possible to identify additional markers associated with the clinical course, the risk of invasion and metastasis, as well as to supplement and refine the existing classification or propose a new one based on the molecular characteristics of the tumor (19–22).

The beginnings of gene study date back to Sanger et al. who introduced his chain termination method for sequencing DNA in 1977 (23), that quickly gained great acceptance and popularity around the world, becoming in fact the first generation of the DNA sequencing technology (24, 25).

Next-generation sequencing(NGS) is a method of sequencing multiple DNA or RNA products in parallel. This technique is also known by other names (eg, short-read sequencing, deep sequencing, second-generation sequencing). In contrast to Sanger sequencing, the speed of sequencing and the amount of DNA sequence data generated by NGS are exponentially higher, and the cost of production is significantly lower (26). The most complete molecular assessment lymphoma genetics was obtained by using whole genome sequencing of all coding sequences (exome) by high-throughput next-generation parallel sequencing (WES). WES studies were performed for each of the major immunomorphological subtypes of lymphomas: DLBCL, Burkitt lymphoma, follicular lymphoma, mantle, splenic marginal zone lymphoma, and peripheral T-cell lymphomas (27).

The contemporary diagnosis of NHLs is based on morphological and immunophenotypic studies, as well as chromosomal and molecular analyses, which are indicated as diagnostic procedures to establish high-precision diagnoses (28). The current recommendations, however, do not provide clear stipulations for the conditions of sequencing techniques used for NHL diagnosis and prognosis. There is no uniform strategy at this time, and aspects such as gene selection, sequencing platform, read depth, and variant analysis may vary among laboratories. Therefore, standardization of the panels is needed especially taking into account the fact that the NGS panels of the lymphoid lineage are becoming more accessible for clinical practice.

The purpose of this article is to provide a comprehensive overview of the gene panels that are identified in different NHL types by the use of NGS techniques.

## 2 B-cell lymphomas

B-cell lymphomas represent the predominant type of NHL diagnosed globally. About 85-90% of NHL cases are derived from B cells, whereas the remaining lymphomas originate from T cells or NK cells (6, 29). This epidemiological circumstance likely explains the greater inclination for studying the genomic and transcriptomic features of these neoplasms by various research groups. In the following sections, we will describe the specifics of gene expression profiles in some of the most common types of NHL. **Table 1** includes a summary of the most common GEP associated with different types of B-cell lymphomas.

**Table 1:** Genetic profile of B cell lymphomas.

Type of lymphoma	Genetic profile	References
Diffuse large B cell lymphoma, GCB subtype	*BCL2/BCL6, EZH2,GNA13, IRF8, MYC, SGK1, STAT3, TNFR14*	(17) (30, 31) (6) (32)
Diffuse large B cell lymphoma, ABC subtype	*CD79b, EP300, KMT2D, MYD88d, PIM1, PRDM1*	(17, 30, 31) (6) (32)
Follicular lymphoma	*DTX1, EP300, EZH2, ARID1A, CREBBP, CARD11, FOXO1*, *HIST1H1E, MEF2B, NOTCH2*, *UBE2A*	(33, 34) (32) (35)
Marginal zone lymphoma	*BTK, NOTCH2, BCL10, BIRC3, CARD11, KLF2, PLCG2, PTPRD*	(32, 36)
Mantle zone lymphoma	*BTK, NOTCH1/2, MALT1, ATM, BCL10, BIRC3, CDKN2A, IKBKB, MAP3K14, NSD2, PLCG2, SMARCA4, TP53, TRAF2*	(37) (38, 39) (32) (40)
Small lymphocytic lymphoma/Chronic lymphocytic leukemia	*ATM, BIRC3, BTK, NOTCH1, PLCG2, POT1, SF3B1, TP53d*	(32, 41) (42)
Primary mediastinal large B-cell lymphoma	*STAT6, XPO1, B2M, NFKBIE, PTPN1,TNFAIP3*	(6, 32) (43)
Burkitt lymphoma	*ID3, TCF3, CCND3, TP53, CDKN2A, MYC, DDX3X, PTEN, PIK3R1, ARID1A, SMARCA4, GNA13, ROCK1*	(6, 32)

### 2.1 Diffuse large B cell lymphoma

Diffuse large B cell lymphoma (DLBCL) is the most frequent type of non-Hodgkin lymphoma in the world, accounting for 30–40% of all occurrences depending on the geographical region (44).

Traditionally, DLBCL cases were classified according to cell-of-origin (COO), with two different subtypes described: germinal center B-cell like (GCB) and activated B-cell like (ABC), and with about 10–15 percent of cases remaining unclassifiable (45).

Patients who have the GCB subtype have a better prognosis than those who have the ABC subtype. Although COO can help predict the outcome, the GCB and ABC subtypes are still very heterogeneous raising the question of a more accurate prognostic stratification (6, 44, 46).

In 2017, Reddy et al. conducted a study that used whole-exome and transcriptome sequencing of tumors from 1,001 newly diagnosed DLBCL patients to determine genetic drivers of the disease and establish probable links to clinical outcomes (47).

As a result, the authors identified 150 genes that are directly involved in the pathogenesis of DLBCL. These genes can be classified into four main categories:

1) genes involved in signaling pathways (for example, *MTOR, PIK3R1, PIM2, BTK*);
2) genes associated with transcription and translation in the cell (for example, *SF3B1, XPO1, HIST1H1E*);
3) genes responsible for the stages of B cell differentiation (for example, *EBF1, IRF4, PAX5, POU2F2, YY1*);
4) genes responsible for cell growth and proliferation (for example, *MYC, CHD8, BCL2*).

Also, *MYD88* was chosen as a critical mutation in the ABC subtype, whereas *XPO1* was chosen as an essential mutation in GCB DLBCL. The publication is limited by the lack of explanation in case of DNA mutation-based disease clustering, focusing only on RNA-based or translocation-based classification with DNA mutations (47).

Chapuy et al. in 2018, proposed a DNA-based classification of DLBCL. In this study WES was performed on 304 patients samples. C1–C5 were the names given to these clusters, permitting the classification of ABC and GCB-DLBCL cases into two different groups with favorable and adverse outcomes. ABC subtypes were divided into two groups: a lower risk group with a putative marginal zone origin (C1) characterized by *NOTCH2* mutations/*BCL6* translocation, and one with a higher risk (C5) with chromosome 18q gain with *BCL2* and *MALT1* gene overexpression and *CD79B* and *MYD88* mutations. The C2 subgroup was associated with biallelic loss or mutation of TP53 and widespread somatic copy number alterations. Additionally, C2 tumors frequently showed copy loss of 9p21.13/CDKN2A and 13q14.2/RB1. Two other subtypes of the GCB were identified (C3 and C4). C4, which was associated with low risk disease and revealed mutations impacting the *BCR/PI3K, JAK/STAT*, and *BRAF* pathways. Conversely, mutations impacting *BCL2* translocation, *PTEN*, and epigenetic mediators such as *KMT2D*, *CREBBP*, and *EZH2* were all linked to the poorer prognosis of the *C3* subgroup (17).

Schmitz et al. used whole-exome and transcriptome sequencing, DNA copy number analysis, and deep targeted amplicon sequencing to examine data from 574 DLBCL patients. As a result, four different subtypes of DLBCL were identified: MCD (based on the presence of *MYD88L265P* and *CD79B* mutations), BN2 (based on *BCL6* fusions and *NOTCH2* mutations), N1 (based on the presence of *NOTCH1* mutations), and EZB (based on the presence of *EZH2* mutations and *BCL2* translocations) (30). There are numerous parallels between Chapuy’s and Schmitz’s subgroups, including the following: C1 resembles the BN2 group, C3 overalps EZB, and C5 is similar to MCD.

**Figure 1:**
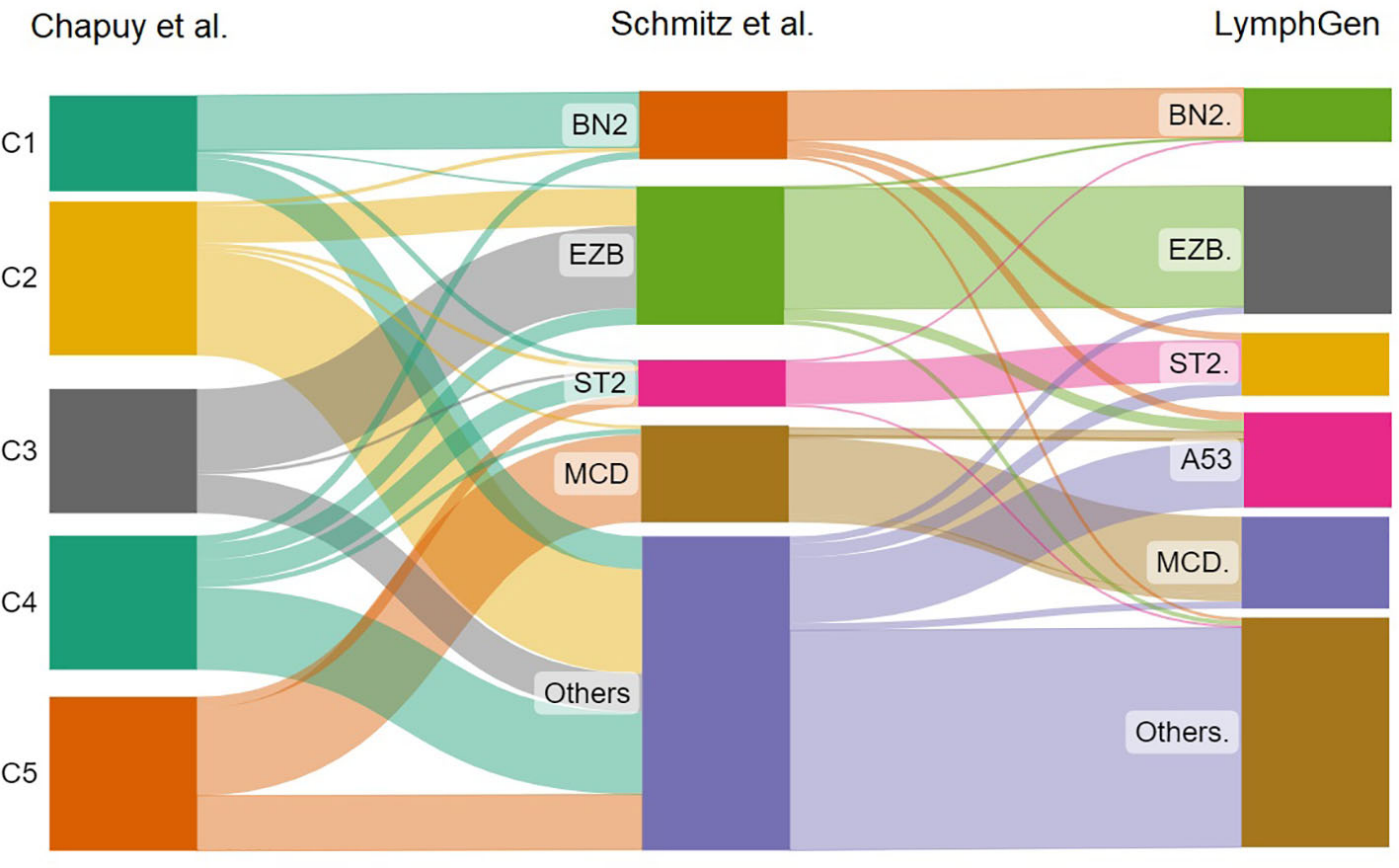
The sankey plot shows the relative proportion of cases from the Chapuy et al. classification that correlates with the Schmitz et al. molecular groups and LymphGen classification.

A follow-up to the findings of Schmitz et al. was the research done by Wright et al. examining the initially unclassified cases. The researchers identified two other subtypes, one with high levels of aneuploidy and mutation of *TP53*, and the second *ST2* (*SGK1* and *TET2* mutations). These corresponded closely to the Chapuy subgroups, C2 and C4. Thus, each of the five Chapuy clusters could now be mapped to one of the Schmitz genetic subgroups. LymphGen is the name given to this classification at the moment (31). The correlation between these three molecular classifications is shown in **Figure 1**.

Recently, studies incorporating clinical, biochemical, and genetic data into multimodal machine learning models have yielded to the elaboration of a gene expression profiling tool that is offering encouraging results in terms of more accurate DLBCL prognostication (48).

### 2.2 Follicular lymphoma

Follicular lymphoma (FL) represents the second most common non-Hodgkin lymphoma and the most prevalent indolent lymphoma. The chromosomal translocation t(14;18)(q32:q21), in which the immunoglobulin heavy chain (*IGH*) enhancer region at 14q32 and the B-cell lymphoma 2 (*BCL2*) gene at 18q21 are juxtaposed, is the hallmark of FL, which is identified in about 90% of cases (49). NGS research has been useful not only in creating a list of genomic events that occur in addition to t(14;18), but also in identifying new potential genetic drivers. The high frequency of mutations affecting epigenetic control is the second distinguishing feature of FL.

Deregulation of such processes (e.g. aberrant DNA hypermethylation) has been recognized as a central feature of hematologic malignancies, and FL in particular, observed in 80% of cases (50). The histone methyltransferases *KMT2D* (90%) and *EZH2* (25%) as well as the histone acetyltransferases *CREBBP* (30–60%) and *EP300* (9%) are among the most commonly mutated genes (51). A clinical-genomic score was created using seven genes including those mentioned above, to predict Failure-Free Survival (FFS) and Overall Survival (OS) (33).

In the era of FL treatment with conventional chemotherapy, the scientists found that mutations in *EP300, FOXO1, CREBBP*, and *CARD11* (providing poor prognosis) and *MEF2B, ARID1A*, and *EZH2* (providing good prognosis) in association with clinical parameters of the FLIPI score, improved PFS and OS prediction. Furthermore, the m7-FLIPI was able to reclassify almost half of the high-risk FLIPI patients into a low-risk m7-FLIPI group, mainly through the discovery of *EZH2* mutations (33, 34). An another research on the m7-FLIPI score across different populations with FL suggests that this molecular score has no impact on patients with FL, treated in the first line, with chemotherapy-free regimens (52, 53). In addition, another study has shown that four mutant genes in FL samples (*NOTCH2, DTX1, UBE2A*, and *HIST1H1E*) were linked to shorter transformation time to DLBCL (35).

In a recent study performed by Gao et al., for the first time, they studied the genomic and transcriptomic characteristics that could predict progression of disease within 24 months (POD24). As a result of this study, they identified genomic markers that are able to predict POD24 in patients with FL. So, HIST1H1D, known as a driver mutation, was significantly correlated with POD24. Furthermore, gains of 6q22.2 (HIST1H1D) and 18q21.33 (BCL2) and loss of 1p36.13 (NBPF1) predicted POD24 independent of FLIPI (54).

### 2.3 Mantle cell lymphoma

Mantle Cell lymphoma (MCL) is an incurable type of aggressive lymphoma with a median survival of approximately 5 years (38, 55). The revised World Health Organization classification from 2016 identified two molecular routes of MCL dividing them in cases of Nodal MCL and Leukemic non-nodal MCL (10-20% of cases, more indolent) (6, 39).

More than 30 years have passed since the first report of the well-known hallmark genetic alteration t (11,14) (q13; q32)/*CCND1::IGH*, which is seen in 95 percent of MCL cases. The result of juxtaposition of heavy-chain immunoglobulin (*IGH*) enhancer region (on 14q32) next to *CCND1* (on 11q13), results in its overexpression of Cyclin D1 (56, 57).

MCL was divided into two categories by the WHO classification: classical MCL and indolent leukemic non-nodal MCL. Indolent leukemic non-nodal MCL is characterized by mutated IGHV and primarily *SOX11* negativity, as well as peripheral blood, bone marrow, and occasionally splenic involvement but no major nodal involvement. Classical MCL is characterised by unmutated or minimally mutated IGHV and mostly *SOX11* positivity (6, 58).

In recent years, genomic techniques have revealed mutations with prognostic implication for MCL. A recent meta-analysis summarized the most common mutations discovered using molecular methods in MCL patients. Among the most common mutant genes were: *ATM* (43.5%) followed by *TP53* (26.8%), *CDKN2A* (23.9%), and *CCND1* (20.2%). Aberrations in *IGH* (38.4%) and *MYC* (20.8%) were also discovered, mostly by cytogenetic techniques. Other prevalent baseline mutations included *NSD2* (15%), *KMT2A* (8.9%), *S1PR1* (8.6%), and *CARD11* (8.5%). The authors propose that a panel of these genes shall be added to NGS panels (55). *CDNK2A* deletion, *ATM, NOTCH1/2, NSD2* mutations were highlighted as markers of poor prognosis. Other mutations were described to have potential diagnostic, therapeutic and predictive role, such as those in *BIRC3, BTK, PLCG2, SMARCA4* and *MAP3K14* (40, 59, 60).

Agarwal et al. discovered genetic patterns that separates responders and nonresponders in a prospective study performed on patients with MCL. ATM mutations were found in the majority of patients who had a complete response, while chromosome 9p21.1-p24.3 loss and/or mutations in SWI-SNF chromatin-remodeling complex components were found in all patients with primary resistance and two-thirds of patients with relapsed disease (61).

*TP53* mutation is another significant indicator of MCL prognosis. Patients with *TP53* mutation were related to the blastoid morphology of MCL, elevated Ki-67, high-risk MIPI, and MIPI-c. When compared to *TP53*-unmutated cases, *TP53* mutations lead to inferior results in terms of response following both induction and autologous stem cell transplantation, as well as shorter PFS (62, 63).

Recently, Yi et al. (37) conducted a WES study on 152 samples of MCL patients, classifying MCL molecularly into 4 distinct clusters (C1-C4). C1 had a 5-year OS of 100% and it was associated with mutant immunoglobulin heavy variable (*IGHV*), *CCND1* mutation, amp(11q13), and active B cell receptor (BCR) signaling. C2 was linked with del(11q)/*ATM* mutations, activation of *NF-kB* and DNA repair pathways, and it was associated with a 5-year OS of 56.7%. C3 was characterized by mutations in *SP140, NOTCH1*, and *NSD2*, as well as downregulation of *BCR* signaling and *MYC* targets, and had a 5-year OS of 47%. C4 included patients with del(17p)/*TP53* mutations, del(13q), and del(9p), as well as active *MYC* pathway and hyperproliferation signatures, and it was associated with a poor prognosis (5-year OS of only 14.2%) (37).

## 3 NK/T-cell lymphomas

Malignant T/NK lymphomas(TNKL) are a distinct group of non-Hodgkin’s lymphomas that account for an estimated 10-15% of the total NHL, with a higher incidence in certain geographic areas (Asia, South America) (64, 65). TNKL, like other malignant proliferative disorders, exhibit genetic instability and chromosomal abnormalities, which combined induce malignant transformation. Therefore, the use of NGS and GEP represent a chance to discover new patterns that can have real prognostic and theranostic impact on TNKL. Despite this, for various reasons, compared to B cell lymphomas, there are fewer reports of the use of NGS, WES, WGS in the case of TNKL. Next, we will attempt to compile the existing data which has genuine prognostic or therapeutic implications. Similarly, data on GEP within distinct TNKLs will be reported in **Table 2** separately.

**Table 2:** Genetic profile of T cell lymphomas.

Type of T/NK non-Hodgkin lymphoma	Genetic profile	References
Angioimmunoblastic T-cell lymphoma	*RHOA, TET2, IDH2, DNMT3A, CD28*	(66) (67, 68)
Adult T-cell leukemia/lymphoma	*PLCG1, PRKCB, CARD11, VAV1, IRF4, FYN, CCR4, CCR7, GATA3, HNRNPA2B1, GPR183, CSNK2A1, CSNK2B*	(69–71)
Extranodal natural killer/T-cell lymphoma, nasal type	*TP53, DDX3X, MGA, STAT3, STAT5B, MLL2, ARID1A, EP300, ASXL3, BCOR, MSN, JAK3, KMT2D*	(72–74)
Intestinal T-cell lymphoma	*STAT5B, SETD2, JAK1, JAK3, STAT3, SOCS1, KRAS, TP53*	(75) (76)
Mycosis fungoides/Sezary Syndrome	*TCR, MYC, TOX, TP53, NCOR1, PTEN, FAS, DNMT3A, USP28, CAAP1, TMEM244, EHD1, MTMR2, RNF123, TOX, BAIAP2, CPN2, GPR128, CAPN12, FIGLA*	(77–79)
Subcutaneous panniculitis-like T-cell lymphoma	*mTOR/AKT/PI3K, HAVCR2*	(80)
Peripheral T- cell lymphoma, NOS	*TP53, CDKN2A, WWOX, ANKRD11, pY-STAT3*	(81)
Breast Implant-Associated Anaplastic Large Cell Lymphoma	*JAK1, STAT3*	(82, 83)

### 3.1 Angioimunoblastic T-cell lymphoma

AITL is a distinct clinicopathologic, and genetic subtype of peripheral T-cell lymphoma (PTCL). AITL is the second most prevalent PTCL subtype worldwide, accounting for 15% to 20% of all PTCL cases, and the most common subtype in the Western world, accounting for more than 30% of all PTCL cases (18, 84, 85).

In 2007, De Leval et al. identified the cell of origin being the T follicular helper cell (TFH) based on the use of gene expression profile studies (86). *CD28* (9.4–11.3%), *DNMT3A* (20–30%), *IDH2* (20–45%), *TET2* (47–83%), and *RHOA* mutations (50–70%) are the most common genetic alterations detected in AITL.

The *RHOA G17V* is the result of a valine substitution for glycine at aminoacid 17, which causes the protein to lose its ability to bind GTP. Furthermore, patients with *RHOA* mutations are thought to have enhanced microvascular density and to exhibit a high number of follicular helper T-cell markers (87). In contrast to other mutations such as *TET2* and *DNMT3A*, which can occur in both tumor and nontumor cells of AITL patients, *RHOA* mutations appear to be limited to tumor cells, indicating that they play an important role in AITL pathogenesis (67, 88).

*TET2* encodes a 2-oxoglutarate/Fe2+–dependent oxygenase that participates in the epigenetic control of gene expression by catalyzing the oxidation of DNA 5-methylcytosine to 5-hydroxymethylcytosine. *TET2* was first described as a tumor suppressor in myeloid neoplasms, but afterward, a high loss of function in *TET2* was identified in PTCL and especially AITL (89–91). *TET2* mutations are also found in hematopoietic cells in a borderline disease called clonal hematopoiesis of indeterminate potential (CHIP), and are associated with the risk of clonal malignancy over time. The fact that not all people with CHIP associated with a *TET2* mutation can develop malignant haemopathy indicates that it is necessary to acquire secondary mutations for the malignant transformation to take place (92, 93). Loss-of-function mutations in *DNMT3A*, a DNA methyltransferase, are common in AITL and frequently co-occur with *TET2* mutations (68). Cooperation between *DNMT3A* and *TET2* mutations has been found to result in malignant transformation in mice models (94).

In the mitochondria, the isocitrate dehydrogenase 2 (*IDH2*) gene normally encodes enzymes that convert isocitrate to alpha-ketoglutarate (2-oxoglutarate, aKG). The neomorphic enzymatic activity of the mutant enzymes catalyzes the conversion of alpha ketoglutarate to 2-hydroxyglutarate (2-HG), an oncometabolite that inhibits the function of the *TET* family of enzymes (68). AITL is the only type of PTCL in which recurrent *IDH2* mutations appear. Mutations in position *R172* of *IDH2* are specific for AITL and typically co-occur with *TET2* mutations (68).

*TET2, DNMT3A*, and *IDH2* mutations occur early in hematopoietic stem cell development, contributing to increased clonal hematopoiesis and greater hematopoietic stem cell self-renewal, but they do not impact T cell differentiation and are therefore considered non-lineage impact mutations, according to a recent review by Yu et al. (91) Late in the T-cell lineage differentiation, mutations in *RHOA, VAV1, VAV::STAP2, CD28, CTLA::CD28, ITK::SYK, PLCy1*, and *TNFRSF21* induce malignant T-cell transformation (91). Considering hypermethylation as the fundamental pathogenetic mechanism of AITL, the use of hypomethylating drugs appears to be a reasonable therapeutic option, and is currently in the clinical trials phase (95, 96).

### 3.2 Mycosis fungoides/Sézary syndrome

The nosological entities known as Mycosis fungoides (MF) and Sézary Syndrome (SS) account for about 75% of all Cutaneous T-cell lymphomas (97). SS is a generalized form of the condition that manifests itself clinically with erythrodermic lesions along with lymph node and blood involvement at onset. MF is a disorder with limited expansion in the skin area, being associated with a good prognosis (77, 98).

The difference in COO can explain the clinical distinctions between MF and SS. MF and SS develop from different subtypes of CD4 + memory T cells; The source cell in the case of MF are T resident memory (Trm) cells exhibiting *CCR4 +/CLA +/L-selectin-/CCR7– (TRM)*, which have a higher tropism to the skin and epithelial barriers, while in the case of SS the COO are T-cell central memory cells (Tcm) that express *CCR4 +/Lselectin +/CCR7+*, and these cells have the ability to migrate between skin, lymph nodes and blood (99).

Recent NGS research in MF/SS has found a high rate of C>T transitions (40–74%), a mutational signature linked to ultraviolet B (UVB) exposure that is uncommon to be seen in other hematological neoplasms (100–102).

Litvinov et al. described 17 genes (*CCL18, CCL26, FYB, T3JAM, MMP12, LEF1, LCK, ITK, GNLY, IL2RA, IL-26, IL-22, CCR4, GTSF1, SYCP1, STAT5A, TOX*) that identified those patients who are at risk of progression and differentiated MF/SS from benign dermatological diseases (103).

The accuracy of diagnosing SS using distinct gene panels has been demonstrated by *Nebozhyn et al.* and *Michel et al.* in two separate papers. *Nebozhyn et al.* used a panel of five genes (STAT4, GATA3, PLS3, CD1D, and TRAIL) that could correctly separate patient samples from controls with 90% accuracy. On the other hand, *Michel et al.* used a signature based on four genes (PLS3, Twist1, CD158k/KIR3DL2, and NKp46) with the ability to separate SS samples from control samples in 100% of cases. They noted that only the Twist1 gene has a diagnostic sensitivity of SS of 91% (104, 105).

The largest retrospective WES evaluation of CTCL to date utilized publicly available sequencing data from nine studies, comprising 220 patients with CTCL, which included 186 SS patients and 25 MF patients (106). This study identified fifty-five putative driver genes and implicated seventeen gene mutations previously not described as being involved in CTCL. These novel mutations target pathways that are involved chromatin remodeling (*BCOR, KDM6A, SMARCB1, TRRAP*), immune surveillance (*CD58, RFXAP*), MAPK signaling (*MAP2K1, NF1*), NF-κB signaling (*PRKCB, CSNK1A1*), PI-3-kinase signaling (*PIK3R1, VAV1*), RHOA/cytoskeleton remodeling (*ARHGEF3*), RNA splicing (*U2AF1*), T-cell receptor signaling (*PTPRN2, RLTPR*), and T-cell differentiation (*RARA*) (106). The *JAK/STAT* pathway, which includes *JAK1, JAK3, STAT3*, and *STAT5B*, is frequently affected by gain-of-function mutations and amplifications in CTCL resulting in the hyperactivation of this signaling pathway (106). Nevertheless, genomic studies in MF/SS do not allow to have a complete picture as in B cell lymphomas on the prognostic stratification of cases or the establishment of molecular classification, this will most likely be the moment of interest for further investigations.

## 4 The use of liquid biopsy in non-Hodgkin lymphomas

Currently, the diagnosis of non-Hodgkin’s lymphoma is based on excisional biopsy of the tumoral tissue. Tissue biopsies, however, are invasive methods of diagnosis with a several disadvantages, such as the risks of tissue biopsy (bleeding, infection, functional disability, etc.), the difficulty of obtaining biopsy samples, and do not allow the dynamic heterogeneity of the case to be assessed (107).

The concept of liquid biopsy which is a non-invasive technique, and can be used to explore the entire mutational landscape of the lymphoma. Liquid biopsy allows for an evaluation of lymphoma at the stage of diagnosis, and prognostic stratification.

Both healthy cells and malignant cells release nucleic acids (DNA, mRNA, and miRNA) into body fluids like the cerebrospinal fluid, peripheral blood, and urine. The term “cell-free DNA” (cfDNA) refers to non-cell-bound DNA fragments discovered in the circulatory system. cfDNA often contains both normal DNA and circulating tumor DNA (ctDNA). The lysis of circulating tumor cells (CTCs), apoptosis, necrosis, or the release of DNA from tumor cells into the bloodstream are possible origins for the tumor-specific part of cfDNA. Because cfDNA can emerge from both malignant and non-malignant cells, assays for the detection of ctDNA are more specific for tumor identification in the case of non-Hodgkin’s lymphomas (108, 109).

Close monitoring of NHL cases by using ctDNA quantification of liquid biopsies can identify the genetic heterogeneities that appear between the primary tumor and the primary areas of metastasis, as well as between various locations of metastases. This information can then be used to find biomarkers indicative of spreading mechanisms and lymphomatous transformation. Multiple studies including a recent meta-analysis, have shown higher levels of cfDNA in cancer patients compared with healthy controls. Different subsets of lymphoma can be distinguished at the time of diagnosis with the help of NGS-based analysis of ctDNA. Furthermore, ctDNA load strongly reflects tumor burden, as it appears to correlate significantly with lactate dehydrogenase (LDH) and the International Prognostic Index (IPI), as observed in DLBCL, NKTCL and other types of lymphomas (110–113). In DLBCL, interim ctDNA monitoring during therapy directly evaluates tumor kinetics response and foretells early treatment failure. The determination of interim levels of ctDNA has a greater sensitivity than existing imaging methods, creating a so-called “window of opportunity” during which, the earlier initiation of salvage therapy prior to clinical relapse to be diagnosed, has the potential to improve outcomes (114).

In the case of DLBCL, initial levels of ctDNA are significantly associated with the International Prognostic Index (IPI), total metabolic tumor volume (TMTV), lactate dehydrogenase (LDH) concentrations, and the Ann Arbor stage. Pretreatment ctDNA concentrations have been demonstrated to be highly accurate predictors of clinical outcomes in univariate and multivariate analysis in those trials, and hence gain prognostic importance (43, 115, 116). ctDNA in DLBCL can also be used for the real-time assessment of treatment response, increases in ctDNA levels and changes in KMT2D mutation status have been found to be useful indicators of disease progression (117). The depth of response is an important predictor of outcomes in the post-treatment surveillance of NHL subtypes. Relapsed NHL likely originates from MRD below the current level of detection, and a recent systematic review demonstrated that between 7% and 20% of DLBCL patients in remission by PET scans will ultimately relapse (118). A recent MRD study on DLBCL patients treated with CAR-T cell therapy has shown better sensitivity and predictive value for progression to treatment than the PET scan (119). This study, among others, suggests that liquid biopsy and NGS would create an excellent platform for assessing the efficacy of treatments (116).

In the case of extranodal natural killer/T cell lymphoma (ENKTCL), a recent study explored the use of ctDNA methylation markers for diagnosing, continuously monitoring, and predicting the prognosis. This research has proposed a score formed by 7 ctDNA markers, namely HLX-AS1, MIR12123, CHST12, DLK1, LINC02115, MIR3973, and NCAM, which achieves over 90% accuracy in distinguishing ENKTCL from nasopharyngeal carcinoma, nasopharyngitis, and normal conditions (120).

However, despite the encouraging data of NHL evaluation by liquid biopsies, few validation studies have been published at the moment (121), with the vast majority of the data presented requiring validation in further research.

## 5 NGS use for a personalized approach and future perspective of use

NHL remains a condition treated primarily whit chemoimmunotherapy. The standard of care for years has been frontline R-CHOP, despite multiple attempts to investigate more aggressive regimens like R-DA-EPOCH or incorporate new therapies like obinutuzumab, bortezomib, or ibrutinib. Frontline R-CHOP cures around 60% of DLBCL cases. Nowadays, DA-EPOCH-R is utilized as the first-line therapy for double/triple hit lymphomas, primary mediastinal B cell lymphoma, and HIV-associated DLBCL.

The discovery by Wilson et al. that the co-occurrence of mutations in *MYD88* and *CD79B* can predict response to ibrutinib is an illustration of possible clinical utility of genomic profile data in DLBCL, that may have a real impact in the practice (122).

In MCL, the data obtained through genome sequencing allowed the identification of a group of patients in whom there are inactivating mutations in the *SWI-SNF* chromatin-remodeling complex that lead to *BCL-XL* upregulation and subsequent resistance to the therapeutic combination with ibrutinib and venetoclax (61).

Many T-cell lymphomas harbor mutations in epigenetic regulatory genes, such as *TET2*, *DNMT3A*, and *IDH2*, but they are most frequently seen in AITL. Therefore, the use of drugs from the class of HDAC inhibitors or demethylating agents may have a potential beneficial role.

Recently Huang et al. have proposed the DrugComboExplorer, a computational systems biology tool that concurrently integrates pharmacogenomics profiles of 5585 drugs and bioactive compounds from the NIH LINCS program (Library of Integrated Network-based Cellular Signatures) and genomic profiles for specific cancer types (i.e., signaling pathways, interactome, and pharmacological data). This tool does large-scale medication combination prediction and integrates multi-omics data from cancer patients including non-Hodgkin lymphomas (123).

In conclusion, the knowledge provided by the genomic mapping of non-Hodgkin’s lymphomas in near future will allow the targeting of molecular pathways that cause treatment refractoriness or, on the contrary, the inhibition of which is vital in stopping uncontrolled tumor proliferation. Personalized medicine will not only select a single mutation that it will inhibit through the action of a drug, but by selecting molecular targets that have a synergistic costimulatory or inhibitory effect thus self-potentiating. The increased interest in this field confirms that the integration of genomic and transcriptomic data will allow a better understanding of the therapy of malignant lymphomas and of tumor resistance.

## Author contributions

VT: Validation, Writing – original draft. AM: Validation, Writing – review & editing. CS: Validation, Writing – original draft. MR: Validation, Writing – original draft. SB: Validation, Writing – original draft. MF: Validation, Writing – review & editing.
